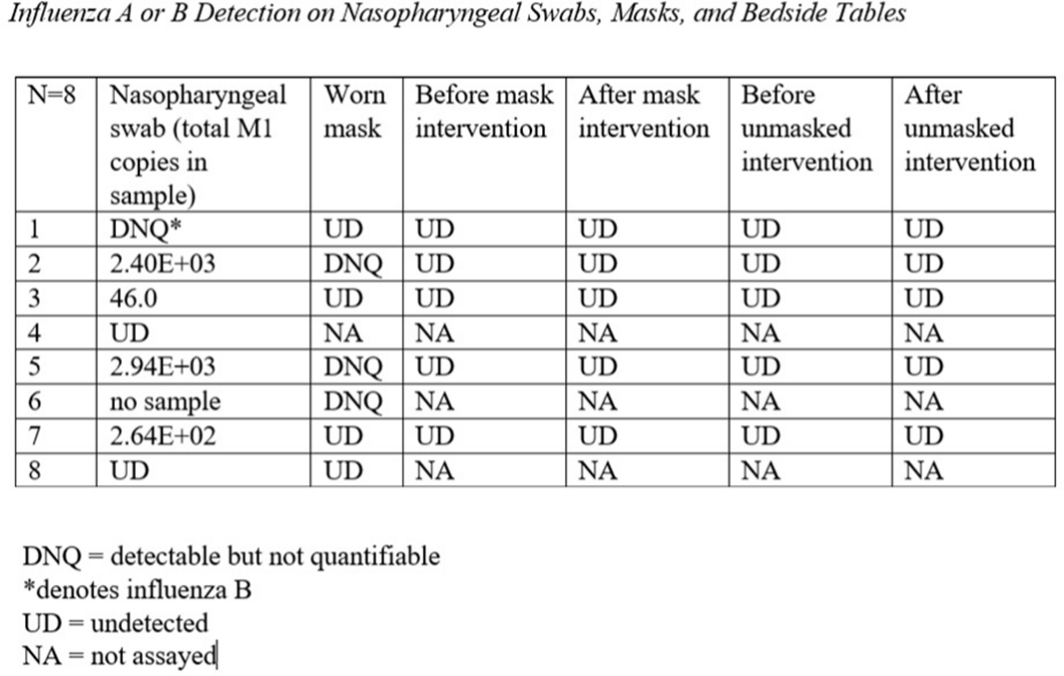# Facemasks for Source Control: Testing Influenza Transfer to Bedside Tables

**DOI:** 10.1017/ash.2021.151

**Published:** 2021-07-29

**Authors:** Adriane Biggio, Stephanie Nagy-Agren

## Abstract

**Background:** Research testing human study participants regarding the effectiveness of face masks in preventing influenza transfer or transmission is limited. In this pilot study, we investigated the following question: In influenza-positive veterans, what is the effect of face-mask wearing in comparison to not wearing a face mask on influenza transfer to bedside tables measured for 2 hours per condition over a 10-week period during the 2019–2020 influenza season **Methods:** Influenza-positive veterans with influenza symptom onset ≤ 120 hours admitted to the Salem Veterans Affairs Medical Center were recruited to participate in this study. Exclusion criteria included critical illness requiring an oxygen mask or intubation. The Precept® FluidGard® 160 Procedure Mask 15300, Precept Medical Products, Inc., Arden, NC was worn by all participants during the two-hour intervention period. Surface swabs were used to measure the presence of influenza on bedside tables. CDC/NIOSH tested for influenza A and B from surface samples and facemasks using real-time polymerase chain reaction (PCR) assay (TaqMan ThermoFisher Scientific). Demographic information was collected (Table 1). A study questionnaire collected qualitative data on tolerability and feasibility of wearing a facemask when hospitalized with influenza. Institutional Review Board approval was granted. **Results:** From January 2, 2020, to March 11, 2020, 8 participants completed the study. Mean age was 67 years, all were male. Of these 8 participants, 6 had influenza A and 2 had influenza B. Half were diabetic; all received oseltamivir. Relative room humidity ranged from 15.6% to 39.8%. Neither influenza A nor B was detected by qPCR on bedside tables for any of the 8 participants under either face-mask–wearing condition. All participants reported that wearing the face mask was easy or very easy; of these, 5 reported experiencing warmth from the mask. Also, 50% of participants selected 2 hours as the time they could tolerate wearing a mask; the other 25% specified they could wear the face mask for 3 hours or 5 hours or more, respectively. **Conclusions:** In this pilot study, we demonstrated that wearing face masks is a tolerable infection control practice for providing source control for inpatients with influenza and will guide future research. Because a major limitation was the small size of the study, associated with lack of viral capture, a larger study is planned. Using face masks for source control among inpatients with influenza and other respiratory virus infections should be considered a standard infection control practice.

**Funding:** No

**Disclosures:** None

**Table 1.**
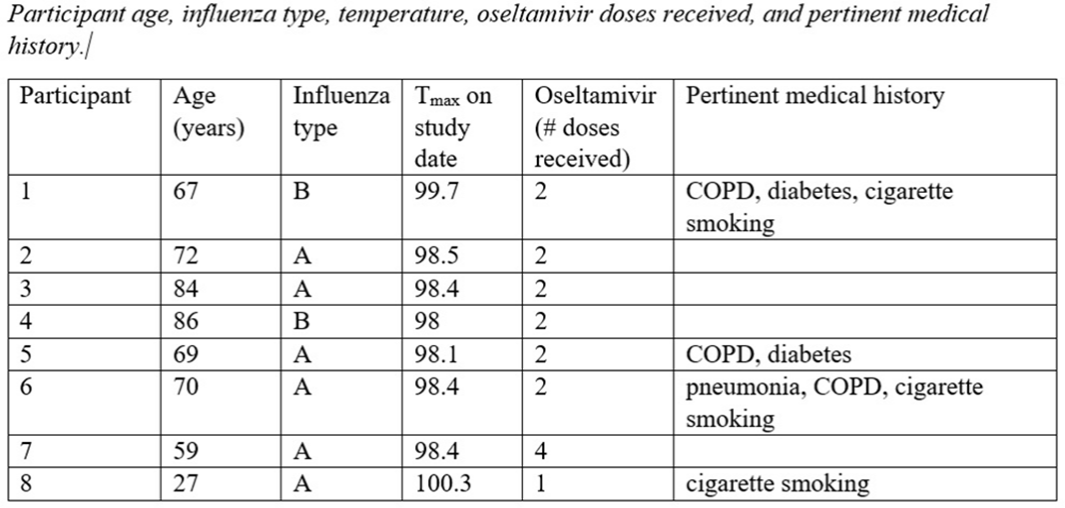

**Table 2.**
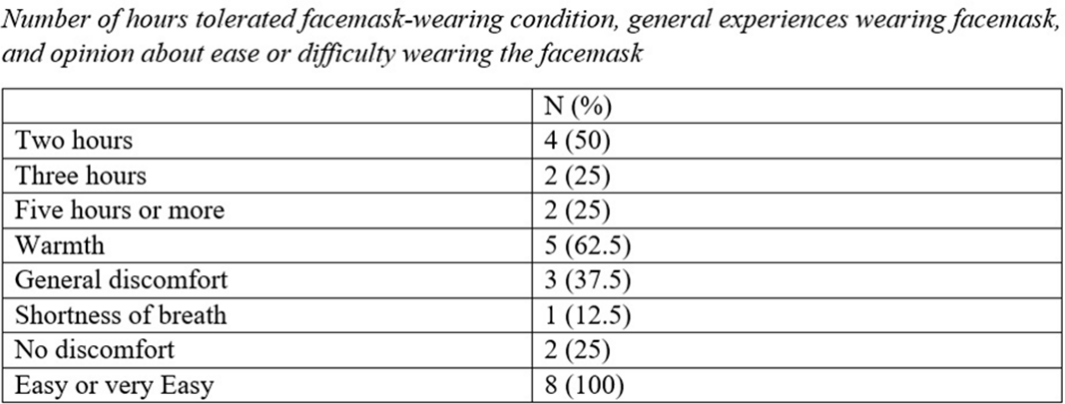

**Table 3.**